# Comments on: involving service users in the qualitative analysis of patient narratives to support healthcare quality improvement

**DOI:** 10.1186/s40900-019-0157-z

**Published:** 2019-09-11

**Authors:** Marney Williams, Mike Etkind, Fran Husson, Della Ogunleye, John Norton

**Affiliations:** 1 Imperial College Healthcare NHS Trust, Fulham Palace Rd, Hammersmith, London, W6 8RF UK; 2 NIHR Imperial Patient Safety Translational Research Centre, London, UK

**Keywords:** Service user involvement, Data analysis, Research

## Abstract

**Plain English summary:**

Some previous researchers (Locock et al) have written about what may be the best way for public contributors to be involved in data analysis in research projects. Their experience has been that giving public contributors large amounts of text to read is not the best use of their time and experience. They have recommended that a better approach would be for a researcher to meet with a group of users at the start of analysis, to discuss what to look out for. However, as another patient group that has been involved in analysis, we think differently. The approach we used was to be more fully involved in the project over a longer time period. Analysis tasks were broken down into stages to make it easier for those taking part. We found that this allowed us to take part fully without placing too much burden on us. We found that our approach was workable and successful and see no reason why it could not be applied in other circumstances.

**Abstract:**

In this journal, Locock et al. have suggested that service users should not be overburdened with large amounts of data, and that eliciting users’ reflections on their experience at the start of analysis and using this as a guide to direct researcher attention during the remainder of the process may work better. As public contributors that have been involved in analysis we suggest an alternative approach in this brief letter, based on our own experiences.

We read the recently published paper on service user involvement with qualitative analysis [1] with interest as we are a public involvement group informing a study about patient-held information about medicines [2] and have recently undertaken analysis of some of the qualitative data from this study. Our group has used a very similar approach to that taken in another previous project [3], cited by Locock et al. We agree with Locock et al. [1] that conversation is at the heart of productive public involvement, but don’t necessarily agree that “conversation rather than data is at the heart of user involvement in analysis”. We feel that asking public contributors to identify touchpoints from their own experience to direct the focus of researchers undertaking analysis occupies an uneasy space between involvement and study participation.

We have not had the difficulties that that Locock et al. [1] encountered, and therefore disagree with their conclusion that the public might struggle to analyse raw data. We remain wedded to the idea that the data should remain at the heart of the analysis and argue that members of the public can realistically and meaningfully contribute to this. We agree with Locock et al’s [1] reflection that they may have been unrealistic in the demands that they placed on their public contributors. Education, training and completion of a novel task is a huge undertaking in 1 day, especially in groups for whom fatigue is a well-known problem. This may have been compounded in a group whose members did not already know each other.

Our involvement group has been active since the start of the study and knows the project well. Group dynamics are understood; we do not need to hear each other’s stories to validate our own experiences. We agreed how we wished to approach the analysis which included the following stages; a 2 h training session on analysis and a particular theory, distribution of anonymised scripts for us to consider at home in our own time, and then a meeting on a later date to discuss the themes found. Notes on our findings were taken by the researcher and subsequently compared with hers. As Locock et al. [1] found, most themes were similar, but with some differences of emphasis.

We would therefore like to suggest that the problems that Locock et al. [1] encountered could be addressed by embedding involvement in analysis into involvement in the project as a whole, and by breaking down the task into sections over a longer time period, rather than dissociating the analysis from the data.

## Data Availability

Not applicable

## References

[1] Locock L, Kirkpatrick S, Brading L, Sturmey G, Cornwell J, Churchill N, et al. Res Involv Engagem. 2019. 10.1186/s40900-018-0133-z. 10.1186/s40900-018-0133-z PMC6318884 30788147

[2] Garfield S, Furniss D, Husson F, et al. Use of patient-held information about medication (PHIMed) to support medicines optimisation: protocol for a mixed-methods descriptive study. BMJ Open. 2018. 10.1136/bmjopen-2018-021764. 10.1136/bmjopen-2018-021764 PMC6042590 29950473

[3] Garfield S, Jheeta S, Husson F, Jacklin A, Bischler C, Norton C, Franklin BD. Lay involvement in the analysis of qualitative data in health services research: a descriptive study. Res Involv Engagem. 2016. 10.1186/s40900-016-0041-z. 10.1186/s40900-016-0041-z PMC5831865 29507764

